# Response to Han et al.

**DOI:** 10.14309/ctg.0000000000000497

**Published:** 2022-05-26

**Authors:** Phil Greer, Dan Spangolo, Celeste Shelton Ohlsen, Mitchell Ellison, Cameron Breze, Mark Haupt, David C. Whitcomb

**Affiliations:** 1 Ariel Precision Medicine, Pittsburgh, Pennsylvania, USA;; 2 University of Pittsburgh Medical Center, Pittsburgh, Pennsylvania, USA;; 3 University of Pittsburgh, Pennsylvania, USA.

On behalf of our team, we are happy to respond to the comments by Han et al. on our recent publication, “Acute and chronic pancreatitis disease prevalence, classification and comorbidities: A cohort-study in the UK Biobank” (1).

First, Han et al. suggested “a more reasonable” grouping of disease states into 3 groups of acute pancreatitis (AP), recurrent acute pancreatitis (RAP), and chronic pancreatitis (CP), rather than showing that it is possible to separate them into 5 groups, including AP + CP, RAP + CP, and CP only (without AP or RAP). We disagree, primarily because of the fact that the number of RAP subjects with no CP (284) is proportionally small compared with the number of subjects with AP and CP. Furthermore, a large proportion of subjects with RAP never progress to CP (2–4). In addition, the ability to distinguish all 5 groups is critical in understanding the mechanisms leading to CP (5,6) because between 16% and 48.5% of all subjects with CP do not have a history of AP (3,4,7,8) and there is currently no mechanistic explanation of CP without AP. Having shown that we can separate out these subpopulations, we hope that future studies may provide insights into alternative mechanisms of CP pathogenesis.

Second, Han et al. objected to the analysis of the incidence of AP and CP increasing based on the assumption that this apparent increase was due to aging of the cohort. However, a careful reading to the article will reveal that we are calculating the “Pancreatitis prevalence and incidence in the UKBB” (page 5, column 1, heading for paragraph 2). Furthermore, in the discussion we state the following: “Several of these studies have shown an increase in incidence with age similar to what we see in the UKBB. Our findings fall well into this range and show a very distinct increase in incidence from 21.4 to 48.2 per 100,000 per year coinciding with the overall aging of our cohort” (page 7, paragraph 2). We believe it is a misreading of our article to conclude that we are generalizing the increase in AP and CP incidence to the general population of the United Kingdom.

Third, Han et al. were critical of our analysis by not including ICD-9 codes for AP and CP. We stand by our decision to limit the comorbidity analysis to ICD-10 based on several facts. First, the ICD-9 data are “only available for older Scottish hospital records” (https://biobank.ndph.ox.ac.uk/showcase/field.cgi?id=41271 under “Notes” tab). The number of Scottish subjects with ICD-9 diagnoses represents only 4.04% of the entire UKBB, and including data from only one section of the United Kingdom may not be representative of the entire UKBB cohort. Second, the effect of excluding ICD-9 codes is small because ICD-9 diagnoses make up less than 1% of the total diagnoses released to date. Third, the ICD-9 codes are limited and lack much of the granularity of the ICD-10 comorbidities. Therefore, the methods for harmonizing ICD-9 and ICD-10 diagnoses may bias the results in categorical analyses. Thus, the use of ICD-10 codes in this cohort is more accurate and more useful for categorical analyses.

Fourth, Han et al. pointed out a typographical error of “283” in the AP group, which should have been 284 (page 4, column 2, line 6). This typo has no impact on any calculations or conclusions, but we thank them for pointing this out.

In conclusion, the UK Biobank is a valuable resource for the study of AP, RAP, and CP and associated factors, although it was not designed specifically to study pancreatic diseases and is limited to a “middle-aged and elder” population. We appreciate the interest of Han et al. in our work and trust that our answers are enlightening. We stand behind both our approach and results.

## CONFLICTS OF INTEREST

**Guarantor of the article**: David C. Whitcomb, MD.

**Specific author contributions**: All authors approved the submitted manuscript.

**Financial support:** None to report.

**Potential competing interests:** P.G. and C.S.O. are employees of Ariel Precision Medicine. M.E. and C.B. are consultants to Ariel Precision Medicine. D.C.W. is a cofounder of Ariel Precision Medicine and a chief scientific officer. D.S. and M.H. are former employees of Ariel Precision Medicine.
